# 9-*O*-Butyl­berberrubinium bromide

**DOI:** 10.1107/S1600536809018388

**Published:** 2009-05-23

**Authors:** Zhu Chen, Xue-Gang Li, Yong-Sheng Xie, Xiao-Li Ye

**Affiliations:** a College of Pharmaceutical Sciences, Southwest University, Chongqing 400715, People’s Republic of China; b School of Chemistry and Chemical Engineering, Shandong University, Jinan 250100, People’s Republic of China; c College of Life Sciences, Southwest University, Chongqing 400715, People’s Republic of China

## Abstract

In the title compound, C_23_H_24_NO_4_^+^·Br^−^, the butyl chain is disordered between two conformations; the occupancies refined to 0.735 (7) and 0.265 (7). The dihedral angle between the naphthalene ring system and the phenyl ring is 11.6 (2)°. In the crystal structure, the cations are packed *via* π–π inter­actions into stacks propagating in the [010] direction. Weak inter­molecular C—H⋯O and C—H⋯Br hydrogen bonds contribute further to the crystal packing stability.

## Related literature

For the bioactivity of berberine, see: Jiang *et al.* (1998 ▶); Kupeli *et al.*. (2002 ▶). For the bioactivity of 9-*O*-butyl-berberrubine bromide, see Ye & Li (2007 ▶).
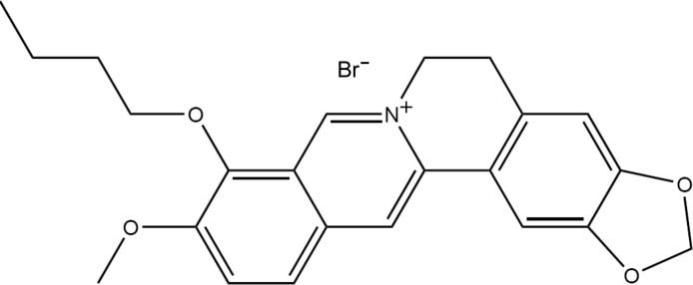


## Experimental

### 

#### Crystal data

C_23_H_24_NO_4_^+^·Br^−^
*M* *_r_* = 458.34
Monoclinic, 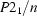

*a* = 9.716 (4) Å
*b* = 7.623 (3) Å
*c* = 27.443 (11) Å
β = 92.983 (8)°
*V* = 2029.9 (14) Å^3^
*Z* = 4
Mo *K*α radiation
μ = 2.06 mm^−1^
*T* = 295 K
0.12 × 0.10 × 0.06 mm

#### Data collection

Bruker SMART CCD area-detector diffractometer
Absorption correction: multi-scan (*SADABS*; Bruker 2005 ▶) *T*_min_ = 0.791, *T*_max_ = 0.887
10394 measured reflections
3592 independent reflections
1965 reflections with *I* > 2σ(*I*)
*R*_int_ = 0.069

#### Refinement

*R*[*F* ^2^ > 2σ(*F* ^2^)] = 0.060
*wR*(*F* ^2^) = 0.161
*S* = 1.08
3592 reflections
276 parameters
H-atom parameters constrained
Δρ_max_ = 0.58 e Å^−3^
Δρ_min_ = −0.55 e Å^−3^

### 

Data collection: *SMART* (Bruker, 2005 ▶); cell refinement: *SAINT* (Bruker, 2005 ▶); data reduction: *SAINT*; program(s) used to solve structure: *SHELXS97* (Sheldrick, 2008 ▶); program(s) used to refine structure: *SHELXL97* (Sheldrick, 2008 ▶); molecular graphics: *XP* in *SHELXTL* (Sheldrick, 2008 ▶); software used to prepare material for publication: *SHELXL97*.

## Supplementary Material

Crystal structure: contains datablocks I, global. DOI: 10.1107/S1600536809018388/cv2550sup1.cif

Structure factors: contains datablocks I. DOI: 10.1107/S1600536809018388/cv2550Isup2.hkl

Additional supplementary materials:  crystallographic information; 3D view; checkCIF report

## Figures and Tables

**Table 1:** Centroid-to-centroid distances (Å)

*Cg*1⋯*Cg*3^i^	3.780 (4)
*Cg*2⋯*Cg*3^ii^	3.775 (4)

Symmetry codes: (i) 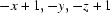; (ii) 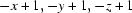. *Cg*1, *Cg*2 and *Cg*3 are the centroids of the N1/C13/C12/C9/C10/C14, C5–C10 and C15/C19–C22/C16 rings, respectively.

**Table 2:** Hydrogen-bond geometry (Å, °)

*D*—H⋯*A*	*D*—H	H⋯*A*	*D*⋯*A*	*D*—H⋯*A*
C12—H12⋯Br1	0.93	2.77	3.658 (5)	161
C2—H2*A*⋯O2^iii^	0.97	2.64	3.404 (14)	136

Symmetry code: (iii) 
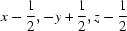
.

**Fig. 1.**
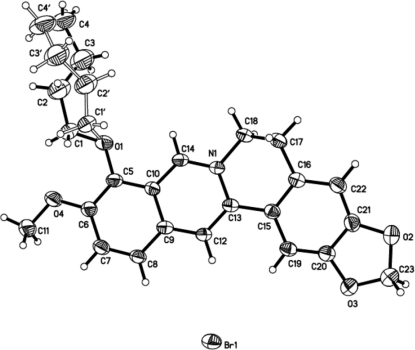
View of the title compound showing the atom-labeling scheme and 30% probability displacement ellipsoids.

## supplementary crystallographic information

### Comment

Berberine is a main compound presented in the rhizome of *Coptis chinensis* Franch. Berberine and its derivatives are used for treating diarrhoea (Kupeli *et al.*, 2002) and anti-inflammatory (Jiang *et al.*, 1998). Herewith we report the crystal structure of the title compound (I), which exhibits an excellent antibacterial activity (Ye & Li, 2007).

In (I) (Fig. 1), the dihedral angles formed by the benzene rings C5–C10 and C15/C19/C20/C21/C22/C16 with the pyridine ring are 2.2 (3) and 12.8 (3)°, respectively. The six-membered heterocyclic ring (C13/C15/C16/C17/C18/N1) adopts screw-boat conformation. In the crystal structure, weak intermolecular C—H···O and C—H···Br hydrogen bonds (Table 2) link the molecules. The aromatic rings in the cations are involved in π–π interactions (Table 1). The cations are packed *via*π–π interactions into stacks propagated in direction [010].

### Experimental

Berberrubine was obtained from berberine by pyrolysis at 180 °C for 1 h. Then dried berberrubine (10 mmol) was dissolved in DMF (200 ml) and incubated with *n*-butyl bromide (11 mmol) for 5 h at 100 °C to give the corresponding crude 9-*O*-butyl-berberrubine bromide. The product was purified by recrystallization from methanol at -18 °C. Crystals suitable for X-ray diffraction were obtained by slow evaporation of a solution of the solid dissolved in methanol at room temperature for 15 d.

### Refinement

All H atoms were placed in calculated positions,with C—H = 0.93–0.97 Å, and included in the final cycles of refinement using a riding model, with *U*_iso_(H) = 1.2Ueq(C) for aryl and methylene H atoms or 1.5Ueq(C) for methyl H atoms.

### Crystal data

**Table:**

C_23_H_24_NO_4_^+^·Br^−^	*F*(000) = 944
*M**_r_* = 458.34	*D*_x_ = 1.500 Mg m^−^^3^
Monoclinic, *P*2_1_/*n*	Mo *K*α radiation, λ = 0.71073 Å
Hall symbol: -P 2yn	Cell parameters from 1163 reflections
*a* = 9.716 (4) Å	θ = 2.8–19.6°
*b* = 7.623 (3) Å	µ = 2.06 mm^−^^1^
*c* = 27.443 (11) Å	*T* = 295 K
β = 92.983 (8)°	Block, yellow
*V* = 2029.9 (14) Å^3^	0.12 × 0.10 × 0.06 mm
*Z* = 4	

### Data collection

**Table:**

Bruker SMART CCD area-detector diffractometer	3592 independent reflections
Radiation source: fine-focus sealed tube	1965 reflections with *I* > 2σ(*I*)
graphite	*R*_int_ = 0.069
φ and ω scans	θ_max_ = 25.1°, θ_min_ = 1.5°
Absorption correction: multi-scan (*SADABS*; Bruker 2005)	*h* = −11→11
*T*_min_ = 0.791, *T*_max_ = 0.887	*k* = −7→9
10394 measured reflections	*l* = −32→32

### Refinement

**Table:**

Refinement on *F*^2^	Secondary atom site location: difference Fourier map
Least-squares matrix: full	Hydrogen site location: inferred from neighbouring sites
*R*[*F*^2^ > 2σ(*F*^2^)] = 0.060	H-atom parameters constrained
*wR*(*F*^2^) = 0.161	*w* = 1/[σ^2^(*F*_o_^2^) + (0.0623*P*)^2^ + 1.1441*P*] where *P* = (*F*_o_^2^ + 2*F*_c_^2^)/3
*S* = 1.08	(Δ/σ)_max_ < 0.001
3592 reflections	Δρ_max_ = 0.58 e Å^−^^3^
276 parameters	Δρ_min_ = −0.55 e Å^−^^3^
0 restraints	Extinction correction: *SHELXL97* (Sheldrick, 2008), Fc^*^=kFc[1+0.001xFc^2^λ^3^/sin(2θ)]^-1/4^
Primary atom site location: structure-invariant direct methods	Extinction coefficient: 0.0020 (7)

### Special details

**Table:**

Geometry. All e.s.d.'s (except the e.s.d. in the dihedral angle between two l.s. planes) are estimated using the full covariance matrix. The cell e.s.d.'s are taken into account individually in the estimation of e.s.d.'s in distances, angles and torsion angles; correlations between e.s.d.'s in cell parameters are only used when they are defined by crystal symmetry. An approximate (isotropic) treatment of cell e.s.d.'s is used for estimating e.s.d.'s involving l.s. planes.
Refinement. Refinement of *F*^2^ against ALL reflections. The weighted *R*-factor *wR* and goodness of fit *S* are based on *F*^2^, conventional *R*-factors *R* are based on *F*, with *F* set to zero for negative *F*^2^. The threshold expression of *F*^2^ > σ(*F*^2^) is used only for calculating *R*-factors(gt) *etc*. and is not relevant to the choice of reflections for refinement. *R*-factors based on *F*^2^ are statistically about twice as large as those based on *F*, and *R*- factors based on ALL data will be even larger.

### Fractional atomic coordinates and isotropic or equivalent isotropic displacement parameters (Å^2^)

**Table:**

	*x*	*y*	*z*	*U*_iso_*/*U*_eq_	Occ. (<1)
Br1	0.19113 (6)	0.34507 (10)	0.58534 (2)	0.0683 (3)	
O1	0.4605 (4)	0.4269 (6)	0.31978 (15)	0.0691 (13)	
O2	0.8340 (4)	−0.0249 (7)	0.66220 (16)	0.0802 (14)	
O3	0.5968 (4)	0.0055 (6)	0.66073 (16)	0.0708 (12)	
O4	0.1935 (4)	0.5291 (6)	0.31514 (15)	0.0741 (13)	
N1	0.6413 (4)	0.2391 (6)	0.44906 (17)	0.0469 (12)	
C1'	0.5253 (10)	0.5997 (11)	0.3125 (3)	0.073 (2)	0.735 (7)
H1'1	0.5814	0.6382	0.3407	0.088*	0.735 (7)
H1'2	0.4589	0.6897	0.3027	0.088*	0.735 (7)
C1	0.4509 (12)	0.5865 (18)	0.2933 (5)	0.073 (2)	0.265 (7)
H1A	0.4497	0.6807	0.3170	0.088*	0.265 (7)
H1B	0.3617	0.5872	0.2757	0.088*	0.265 (7)
C2	0.5550 (11)	0.6342 (14)	0.2577 (4)	0.107 (3)	0.735 (7)
H2A	0.5042	0.6720	0.2282	0.128*	0.735 (7)
H2B	0.6033	0.7367	0.2708	0.128*	0.735 (7)
C3	0.6599 (11)	0.5126 (15)	0.2427 (4)	0.117 (3)	0.735 (7)
H3A	0.6196	0.3962	0.2410	0.140*	0.735 (7)
H3B	0.7335	0.5103	0.2679	0.140*	0.735 (7)
C4	0.7234 (15)	0.5492 (18)	0.1943 (4)	0.120 (4)	0.735 (7)
H4A	0.7620	0.4430	0.1822	0.180*	0.735 (7)
H4B	0.7948	0.6357	0.1990	0.180*	0.735 (7)
H4C	0.6537	0.5922	0.1713	0.180*	0.735 (7)
C2'	0.611 (3)	0.540 (3)	0.2709 (9)	0.107 (3)	0.265 (7)
H2'1	0.6905	0.4764	0.2843	0.128*	0.265 (7)
H2'2	0.5562	0.4586	0.2506	0.128*	0.265 (7)
C3'	0.659 (3)	0.687 (3)	0.2392 (8)	0.117 (3)	0.265 (7)
H3'1	0.7435	0.7352	0.2543	0.140*	0.265 (7)
H3'2	0.5904	0.7792	0.2382	0.140*	0.265 (7)
C4'	0.686 (5)	0.633 (5)	0.1874 (8)	0.120 (4)	0.265 (7)
H4'1	0.7240	0.7304	0.1705	0.180*	0.265 (7)
H4'2	0.6009	0.5976	0.1709	0.180*	0.265 (7)
H4'3	0.7498	0.5369	0.1880	0.180*	0.265 (7)
C5	0.3838 (6)	0.4217 (8)	0.3608 (2)	0.0544 (14)	
C6	0.2479 (6)	0.4712 (8)	0.3590 (2)	0.0570 (15)	
C7	0.1715 (6)	0.4554 (8)	0.4011 (2)	0.0592 (16)	
H7	0.0792	0.4880	0.3995	0.071*	
C8	0.2291 (6)	0.3941 (8)	0.4439 (2)	0.0533 (15)	
H8	0.1758	0.3835	0.4710	0.064*	
C9	0.3696 (5)	0.3464 (8)	0.4475 (2)	0.0482 (13)	
C10	0.4459 (5)	0.3590 (8)	0.4044 (2)	0.0479 (13)	
C11	0.0506 (6)	0.5753 (10)	0.3118 (2)	0.080 (2)	
H11A	−0.0039	0.4744	0.3190	0.121*	
H11B	0.0260	0.6157	0.2794	0.121*	
H11C	0.0339	0.6668	0.3348	0.121*	
C12	0.4368 (5)	0.2854 (7)	0.4901 (2)	0.0482 (14)	
H12	0.3883	0.2809	0.5184	0.058*	
C13	0.5723 (5)	0.2312 (7)	0.4923 (2)	0.0460 (13)	
C14	0.5833 (5)	0.3019 (7)	0.4082 (2)	0.0488 (14)	
H14	0.6356	0.3086	0.3808	0.059*	
C15	0.6459 (5)	0.1642 (7)	0.5359 (2)	0.0476 (13)	
C16	0.7894 (5)	0.1470 (8)	0.5374 (2)	0.0507 (14)	
C17	0.8631 (6)	0.2081 (8)	0.4940 (2)	0.0573 (16)	
H17A	0.8781	0.3337	0.4964	0.069*	
H17B	0.9524	0.1514	0.4938	0.069*	
C18	0.7835 (5)	0.1681 (8)	0.4478 (2)	0.0567 (15)	
H18A	0.7797	0.0421	0.4429	0.068*	
H18B	0.8292	0.2195	0.4206	0.068*	
C19	0.5729 (6)	0.1186 (7)	0.5774 (2)	0.0508 (14)	
H19	0.4775	0.1284	0.5772	0.061*	
C20	0.6445 (6)	0.0617 (8)	0.6165 (2)	0.0532 (15)	
C21	0.7863 (6)	0.0423 (8)	0.6182 (2)	0.0597 (15)	
C22	0.8615 (6)	0.0853 (8)	0.5790 (2)	0.0580 (15)	
H22	0.9569	0.0738	0.5803	0.070*	
C23	0.7160 (7)	−0.0309 (10)	0.6915 (2)	0.0759 (19)	
H23A	0.7082	−0.1461	0.7061	0.091*	
H23B	0.7252	0.0554	0.7174	0.091*	

### Atomic displacement parameters (Å^2^)

**Table:**

	*U*^11^	*U*^22^	*U*^33^	*U*^12^	*U*^13^	*U*^23^
Br1	0.0502 (4)	0.0849 (6)	0.0714 (5)	−0.0009 (4)	0.0186 (3)	0.0016 (4)
O1	0.066 (3)	0.088 (3)	0.055 (3)	0.020 (2)	0.017 (2)	−0.005 (2)
O2	0.069 (3)	0.111 (4)	0.060 (3)	0.010 (3)	−0.007 (3)	0.011 (3)
O3	0.067 (3)	0.088 (3)	0.058 (3)	0.000 (3)	0.006 (2)	0.010 (2)
O4	0.051 (3)	0.116 (4)	0.056 (3)	0.018 (3)	0.003 (2)	−0.001 (3)
N1	0.039 (2)	0.056 (3)	0.047 (3)	0.002 (2)	0.010 (2)	0.000 (2)
C1'	0.077 (4)	0.080 (4)	0.064 (4)	0.002 (4)	0.020 (4)	0.015 (4)
C1	0.077 (4)	0.080 (4)	0.064 (4)	0.002 (4)	0.020 (4)	0.015 (4)
C2	0.117 (6)	0.108 (6)	0.098 (5)	0.002 (5)	0.034 (5)	0.020 (5)
C3	0.133 (6)	0.114 (6)	0.107 (6)	0.002 (6)	0.039 (5)	0.015 (5)
C4	0.140 (9)	0.132 (10)	0.092 (7)	−0.003 (8)	0.061 (6)	0.002 (7)
C2'	0.117 (6)	0.108 (6)	0.098 (5)	0.002 (5)	0.034 (5)	0.020 (5)
C3'	0.133 (6)	0.114 (6)	0.107 (6)	0.002 (6)	0.039 (5)	0.015 (5)
C4'	0.140 (9)	0.132 (10)	0.092 (7)	−0.003 (8)	0.061 (6)	0.002 (7)
C5	0.052 (3)	0.059 (3)	0.054 (3)	0.005 (3)	0.016 (3)	−0.002 (3)
C6	0.051 (3)	0.071 (4)	0.049 (3)	0.004 (3)	0.004 (3)	−0.002 (3)
C7	0.044 (3)	0.074 (4)	0.060 (4)	0.007 (3)	0.005 (3)	−0.005 (3)
C8	0.041 (3)	0.066 (4)	0.054 (3)	0.003 (3)	0.010 (3)	−0.003 (3)
C9	0.044 (3)	0.052 (3)	0.049 (3)	−0.001 (3)	0.011 (3)	−0.008 (3)
C10	0.043 (3)	0.051 (3)	0.050 (3)	0.005 (3)	0.008 (3)	−0.002 (3)
C11	0.061 (4)	0.114 (6)	0.065 (5)	0.016 (4)	−0.003 (4)	0.005 (4)
C12	0.044 (3)	0.055 (3)	0.047 (3)	0.001 (3)	0.012 (3)	0.000 (3)
C13	0.040 (3)	0.049 (3)	0.049 (3)	−0.004 (2)	0.010 (3)	−0.005 (3)
C14	0.043 (3)	0.058 (3)	0.047 (3)	−0.001 (3)	0.014 (3)	−0.004 (3)
C15	0.043 (3)	0.046 (3)	0.055 (3)	−0.003 (3)	0.006 (3)	0.001 (3)
C16	0.044 (3)	0.053 (3)	0.056 (3)	0.001 (3)	0.007 (3)	−0.004 (3)
C17	0.041 (3)	0.064 (4)	0.068 (4)	0.007 (3)	0.011 (3)	−0.001 (3)
C18	0.043 (3)	0.066 (4)	0.062 (4)	0.011 (3)	0.014 (3)	−0.001 (3)
C19	0.041 (3)	0.055 (3)	0.057 (3)	−0.005 (3)	0.007 (3)	−0.006 (3)
C20	0.048 (3)	0.058 (4)	0.053 (4)	−0.003 (3)	0.004 (3)	−0.010 (3)
C21	0.053 (3)	0.066 (4)	0.059 (4)	0.001 (3)	−0.006 (3)	0.001 (3)
C22	0.044 (3)	0.068 (4)	0.063 (4)	0.003 (3)	0.003 (3)	−0.004 (3)
C23	0.074 (4)	0.094 (5)	0.060 (4)	0.005 (4)	0.005 (4)	−0.001 (4)

### Geometric parameters (Å, °)

**Table:**

O1—C5	1.382 (6)	C4'—H4'3	0.9600
O1—C1	1.418 (12)	C5—C6	1.372 (8)
O1—C1'	1.477 (8)	C5—C10	1.397 (8)
O2—C21	1.370 (7)	C6—C7	1.412 (8)
O2—C23	1.434 (7)	C7—C8	1.358 (8)
O3—C20	1.389 (7)	C7—H7	0.9300
O3—C23	1.424 (7)	C8—C9	1.412 (7)
O4—C6	1.361 (7)	C8—H8	0.9300
O4—C11	1.430 (7)	C9—C12	1.389 (7)
N1—C14	1.319 (7)	C9—C10	1.431 (7)
N1—C13	1.393 (6)	C10—C14	1.403 (7)
N1—C18	1.486 (6)	C11—H11A	0.9600
C1'—C2'	1.517 (8)	C11—H11B	0.9600
C1'—H1'1	0.9700	C11—H11C	0.9600
C1'—H1'2	0.9700	C12—C13	1.378 (7)
C1—C2	1.487 (7)	C12—H12	0.9300
C1—H1A	0.9700	C13—C15	1.456 (8)
C1—H1B	0.9700	C14—H14	0.9300
C2—C3	1.454 (7)	C15—C16	1.399 (7)
C2—H2A	0.9700	C15—C19	1.416 (7)
C2—H2B	0.9700	C16—C22	1.391 (8)
C3—C4	1.517 (7)	C16—C17	1.497 (7)
C3—H3A	0.9700	C17—C18	1.483 (8)
C3—H3B	0.9700	C17—H17A	0.9700
C4—H4A	0.9600	C17—H17B	0.9700
C4—H4B	0.9600	C18—H18A	0.9700
C4—H4C	0.9600	C18—H18B	0.9700
C2'—C3'	1.510 (8)	C19—C20	1.321 (8)
C2'—H2'1	0.9700	C19—H19	0.9300
C2'—H2'2	0.9700	C20—C21	1.384 (8)
C3'—C4'	1.516 (8)	C21—C22	1.370 (8)
C3'—H3'1	0.9700	C22—H22	0.9300
C3'—H3'2	0.9700	C23—H23A	0.9700
C4'—H4'1	0.9600	C23—H23B	0.9700
C4'—H4'2	0.9600		
			
Cg1···Cg3^i^	3.780 (4)	Cg2···Cg3^ii^	3.775 (4)
			
C5—O1—C1	114.8 (6)	C8—C7—H7	119.0
C5—O1—C1'	112.8 (5)	C6—C7—H7	119.0
C21—O2—C23	105.2 (5)	C7—C8—C9	120.1 (5)
C20—O3—C23	106.2 (5)	C7—C8—H8	119.9
C6—O4—C11	117.9 (5)	C9—C8—H8	119.9
C14—N1—C13	122.5 (5)	C12—C9—C8	123.7 (5)
C14—N1—C18	117.9 (4)	C12—C9—C10	118.5 (5)
C13—N1—C18	119.6 (5)	C8—C9—C10	117.8 (5)
O1—C1'—C2'	95.0 (10)	C5—C10—C14	122.4 (5)
O1—C1'—H1'1	112.7	C5—C10—C9	120.8 (5)
C2'—C1'—H1'1	112.7	C14—C10—C9	116.7 (5)
O1—C1'—H1'2	112.7	O4—C11—H11A	109.5
C2'—C1'—H1'2	112.7	O4—C11—H11B	109.5
H1'1—C1'—H1'2	110.2	H11A—C11—H11B	109.5
O1—C1—C2	121.2 (9)	O4—C11—H11C	109.5
O1—C1—H1A	107.0	H11A—C11—H11C	109.5
C2—C1—H1A	107.0	H11B—C11—H11C	109.5
O1—C1—H1B	107.0	C13—C12—C9	122.8 (5)
C2—C1—H1B	107.0	C13—C12—H12	118.6
H1A—C1—H1B	106.8	C9—C12—H12	118.6
C3—C2—C1	122.8 (8)	C12—C13—N1	116.9 (5)
C3—C2—H2A	106.6	C12—C13—C15	124.6 (5)
C1—C2—H2A	106.6	N1—C13—C15	118.6 (5)
C3—C2—H2B	106.6	N1—C14—C10	122.5 (5)
C1—C2—H2B	106.6	N1—C14—H14	118.7
H2A—C2—H2B	106.6	C10—C14—H14	118.7
C2—C3—C4	117.0 (8)	C16—C15—C19	119.6 (5)
C2—C3—H3A	108.0	C16—C15—C13	120.2 (5)
C4—C3—H3A	108.0	C19—C15—C13	120.2 (5)
C2—C3—H3B	108.0	C22—C16—C15	121.0 (5)
C4—C3—H3B	108.0	C22—C16—C17	121.2 (5)
H3A—C3—H3B	107.3	C15—C16—C17	117.7 (5)
C3—C4—H4A	109.5	C18—C17—C16	111.6 (5)
C3—C4—H4B	109.5	C18—C17—H17A	109.3
H4A—C4—H4B	109.5	C16—C17—H17A	109.3
C3—C4—H4C	109.5	C18—C17—H17B	109.3
H4A—C4—H4C	109.5	C16—C17—H17B	109.3
H4B—C4—H4C	109.5	H17A—C17—H17B	108.0
C3'—C2'—C1'	114.1 (9)	C17—C18—N1	110.4 (5)
C3'—C2'—H2'1	108.7	C17—C18—H18A	109.6
C1'—C2'—H2'1	108.7	N1—C18—H18A	109.6
C3'—C2'—H2'2	108.7	C17—C18—H18B	109.6
C1'—C2'—H2'2	108.7	N1—C18—H18B	109.6
H2'1—C2'—H2'2	107.6	H18A—C18—H18B	108.1
C2'—C3'—C4'	114.3 (10)	C20—C19—C15	118.0 (5)
C2'—C3'—H3'1	108.7	C20—C19—H19	121.0
C4'—C3'—H3'1	108.7	C15—C19—H19	121.0
C2'—C3'—H3'2	108.7	C19—C20—C21	123.0 (6)
C4'—C3'—H3'2	108.7	C19—C20—O3	128.7 (5)
H3'1—C3'—H3'2	107.6	C21—C20—O3	108.3 (6)
C3'—C4'—H4'1	109.5	O2—C21—C22	127.7 (6)
C3'—C4'—H4'2	109.5	O2—C21—C20	111.1 (6)
H4'1—C4'—H4'2	109.5	C22—C21—C20	121.2 (6)
C3'—C4'—H4'3	109.5	C21—C22—C16	117.3 (5)
H4'1—C4'—H4'3	109.5	C21—C22—H22	121.3
H4'2—C4'—H4'3	109.5	C16—C22—H22	121.3
C6—C5—O1	121.4 (6)	O3—C23—O2	108.1 (5)
C6—C5—C10	119.8 (5)	O3—C23—H23A	110.1
O1—C5—C10	118.8 (5)	O2—C23—H23A	110.1
O4—C6—C5	116.8 (5)	O3—C23—H23B	110.1
O4—C6—C7	123.7 (5)	O2—C23—H23B	110.1
C5—C6—C7	119.5 (6)	H23A—C23—H23B	108.4
C8—C7—C6	121.9 (5)		

Symmetry codes: (i) −*x*+1, −*y*, −*z*+1; (ii) −*x*+1, −*y*+1, −*z*+1.

### Hydrogen-bond geometry (Å, °)

**Table:**

*D*—H···*A*	*D*—H	H···*A*	*D*···*A*	*D*—H···*A*
C12—H12···Br1	0.93	2.77	3.658 (5)	161
C2—H2A···O2^iii^	0.97	2.64	3.404 (14)	136

Symmetry codes: (iii) *x*−1/2, −*y*+1/2, *z*−1/2.
